# Assessing the Feasibility of Developing *in vivo* Neuroprobes for Parallel Intracellular Recording and Stimulation: A Perspective

**DOI:** 10.3389/fnins.2021.807797

**Published:** 2022-01-25

**Authors:** Micha E. Spira, Hadas Erez, Aviv Sharon

**Affiliations:** ^1^Department of Neurobiology, The Alexander Silberman Institute of Life Science, The Hebrew University of Jerusalem, Jerusalem, Israel; ^2^The Charles E. Smith Family and Prof. Joel Elkes Laboratory for Collaborative Research in Psychobiology, The Hebrew University of Jerusalem, Jerusalem, Israel; ^3^The Harvey M. Kruger Family Center for Nanoscience, The Hebrew University of Jerusalem, Jerusalem, Israel

**Keywords:** neural engineering, neuro-electronics, intracellular neuroimplant, microelectrodes, ultrastructure, immunohistology, polyimide

## Abstract

Developing novel neuroprobes that enable parallel multisite, long-term intracellular recording and stimulation of neurons in freely behaving animals is a neuroscientist’s dream. When fulfilled, it is expected to significantly enhance brain research at fundamental mechanistic levels including that of subthreshold signaling and computations. Here we assess the feasibility of merging the advantages of *in vitro* vertical nanopillar technologies that support intracellular recordings with contemporary concepts of *in vivo* extracellular field potential recordings to generate the dream neuroprobes that read the entire electrophysiological signaling repertoire.

## Introduction

The ambitious goal of neuroscience research is to decipher the mechanisms underlying mammalian brain functions during behavior, sensation, learning, memory, cognition, and pathological conditions. This bold objective requires the merging of highly coordinated multidisciplinary and multi-level studies of brain function in freely behaving animals. Understanding brain function on mechanistic levels can be significantly enhanced by developing new tools that monitor the entire electrophysiological brain signaling repertoire in real time and under *in vivo* conditions.

Among the diverse and complementary brain research tools (from molecular to behavioral), we focus here on electrophysiological technology that enables to interrogate the entire biophysical signaling repertoire of neuronal communication and computations at suitable spatiotemporal resolutions in freely behaving organisms.

**FIGURE 1:**
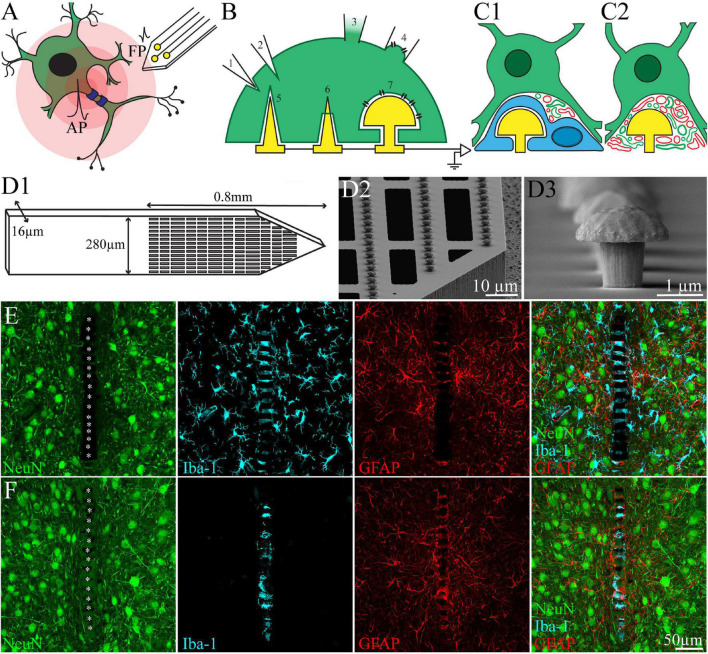
**(A)** Schematic drawing of extracellular field potentials (FPs) recording by implanted neuroprobe. FPs (in the form of first time derivative of an action potential- AP) generated by a neuron (green) are attenuated across the extracellular spaces (centripetal pink shells). Extracellular positioned neuroimplants (yellow) sense and record the FPs but are “blind” to the rich background of subthreshold synaptic potentials that communicate between neurons and play major roles in neuroplasticity. **(B)** Schematic drawing of neuro-technologies that enable to record the entire brains electrophysiological signaling repertoire. **(B1,B2)** Depicting a sharp glass-microelectrode that pierces the plasma membrane of a neuron to gain intracellular accesses. **(B3)** A cell attached patch electrode forming a GΩ seal with the raptured plasma membrane to gain intracellular access. **(B4)** A perforated cell attached configuration in which the plasma membrane is sucked into the patch electrodes and the sucked membrane is nano-perforated to increase its conductance. **(B5–B7)** Schematic drawing of *in-vitro* vertical naopillars for parallel intracellular recording from cultured excitable cells. **(B5,B6)** A sharp vertical nanopillar engulfed **(B5)** and pierces the cell’s plasma membrane **(B6)**. **(B7)** An engulfed mushroom-shaped vertical microelectrode forms an analog configuration to that of the perforated patch electrode shown in **(B4)**. **(C1,C2)** schematic drawings of the biological structures that impede the application of the vertical nanopillar technologies to *in-vivo* brain conditions. **(C1)** A microglia (cyan) form a high impedance seal over the electrode, mechanically isolate and electrically insulate it from the neurons (green). **(C2)** Regenerating neurites (green) and branches of astrocytes (red) occupy the space between the neuron and the microelectrodes. The formed space shunts a large fraction of the current generated by the neuron. **(D1)** Schematics of the polyimide based perforated MEA platform (PPMP), the proximal solid part and distal perforated part are shown. **(D2)** SEM image showing the perforations of the polyimide platform and the dense rows of gMμEs along the PI “ribs”. **(D3)** SEM image of a gMμE. **(E,F)** Identification of a microglia that tightly adhere to the PI platform and insulate the electrodes even after the elimination of the microglia population from the parenchyma by the CSF1R inhibitor PLX5622. **(E)** Control, **(F)** PLX5622 treated rat. Shown are immunohistological sections of the implants along with the cortical parenchyma. Note the distribution of neurons (green), microglia (cyan), and astrocytes (red), around the PPMP implant. For purposes of orientation, the solid PI “ridges” in between the pores of the PPMP are labeled by white asterisks. **(F)** In PLX5622 fed rats the cortex is 95% depleted of microglia. However a PLX5622 resistant microglia population remains adhering to the PPMP and insulates the electrodes. Modified with permission from Sharon et al. (2021a,b).

Currently, two classes of electrophysiological tools are used: (a) Intracellular sharp or patch electrodes that enable analyzing the entire electrophysiological signaling repertoire of a neuron, including excitatory and inhibitory synaptic potentials (EPSPs and IPSPs), membrane potential oscillations, action potential (AP) shapes, “resting membrane potential” and input resistance of individual neurons (Figures 1A,B), and (b), extracellular microelectrodes that record field potentials (FP) generated by propagating APs along single or multiple neurons (Figures 1A,B) and slow potentials involving astrocytes. Whereas intracellular recordings and stimulation are mainly used for biophysical analysis of the elementary mechanisms of neuro-computations and communication of individual neurons or even of sub-neuronal compartments (dendrites or spines), they are severely limited to sampling of single neurons at a time and for a limited durations (approximately 1 h). This is insufficient to study long-term neuroplasticity as in learning, memory and neuropathological processes. In contrast, extracellular microelectrode array (MEA) implants are designed to enable parallel, long-term FP recording from hundreds of distributed neurons. However, extracellular MEAs are unable to record subthreshold IPSPs and EPSPs generated by individual neurons or membrane oscillations. Also, they cannot directly follow meaningful changes in AP shape. As a consequence, neurons that do not fire APs go unnoticed even if they contribute to information processing and computations. Furthermore, parallel extracellular recordings of FP firing patterns are insufficient to directly uncover which neurons receive excitatory or inhibitory synaptic inputs and whether these inputs are altered in the course of learning, memory acquisition or pathological processes. In addition, extracellular FP recordings by implanted MEA platforms suffer from limitations of low signal-to-noise ratio, low source resolution, deterioration of the recording yield and FP amplitudes within days to weeks after implantation (Jackson and Fetz, 2007; Perge et al., 2013; Voigts et al., 2013; Harris et al., 2016; Prodanov and Delbeke, 2016; Lee et al., 2018, 2021).

Regardless of the unequivocal documentation of the critical role of the subthreshold signaling repertoire in normal or pathological brain functions and the technical limitation of extracellular MEA platforms, to our knowledge no attempts to solve the technological deficiency of extracellular recordings have yet been published. Rather, it appears that the *in vivo* neuroengineering/neuroscience communities are focusing their efforts on developing high density extracellular MEA for *in vivo* use. These efforts led to very successful realization of MEA platforms carrying thousands of high-density, small diameter low impedance and addressable electrodes (for example, Jackel et al., 2017; Jun et al., 2017; Raducanu et al., 2017; Viswam et al., 2017; Dorigo et al., 2018; Angotzi et al., 2019; Steinmetz et al., 2021) or the development of highly efficient robotic tools to implant thousands of individual electrodes (Musk, 2019). In spite of the immense progress, the new generations of dense extracellular *in vivo* MEAs relies on the use of planar electrodes and suffers from the innate limitations of being “blind” to subthreshold electrophysiological signals, low signal-to-noise ratio, low source resolution and deterioration of the recording yield and FP amplitudes within days to weeks after implantation.

In parallel to the development of high-density extracellular *in vivo* MEA platforms, a number of investigators have begun to develop *in vitro* MEAs for multisite, intracellular recordings and stimulation from many individual neurons and cardiomyocytes in culture. These *in vitro* MEA technologies utilize different forms of 3D vertical nanostructures (vertical nanopillars, with tip diameters ranging between 50 and 1,000 nm and height of up to ∼6 μm; Figures 1B5–7). These pierce the plasma membrane of cultured cells, like classical sharp glass electrodes, to record attenuated action and synaptic potentials (Figures 1B5,6; Tian et al., 2010; Duan et al., 2012; Gao et al., 2012; Robinson et al., 2012; Xie et al., 2012; Angle et al., 2014; Lin and Cui, 2014; Lin et al., 2014; Qing et al., 2014; Abbott et al., 2017, 2018, 2019; Dipalo et al., 2017; Liu et al., 2017; Li et al., 2020; Yoo et al., 2020; Mariano et al., 2021; Xu et al., 2021; Zhang et al., 2021). Another type of 3D vertical microelectrode are gold mushroom-shaped microelectrodes (gMμEs) that are tightly engulfed by neurons or cardiomyocytes enabling recording of attenuated synaptic and action potentials, while the electrode maintains its extracellular position (Spira et al., 2007, 2018, 2019; Hai et al., 2010a,b; Fendyur and Spira, 2012; Santoro et al., 2013, 2014; Spira and Hai, 2013; Rabieh et al., 2016; Shmoel et al., 2016; Weidlich et al., 2017; McGuire et al., 2018; Mateus et al., 2019; Jones et al., 2020; Teixeira et al., 2020).

Developing novel neuroprobes that enable parallel multisite, long-term intracellular recording and stimulation of neurons in freely behaving animals is the electrophysiologist’s “dream fantasy.” When fulfilled, it is expected to significantly enhance brain research at the mechanistic levels of understanding. Here we begin to assess the feasibility of merging the advantages of *in vitro* vertical nanopillar technologies with concepts of contemporary *in vivo* technologies to generate neuroprobes for parallel intracellular recording and stimulation from many neurons in brains of freely behaving animals.

It is well documented that the quality and yield of recordings by brain neuroprobes reflect complex abiotic and biotic parameters, including the materials from which the probes are constructed, their microarchitecture, sizes, shapes, their surfaces morphology and surface chemistry, the brain regions in which the probe is implanted and the organism used in the study. Here we examine some of the major hurdles to applying the 3D pillar technology, at biotic/abiotic interfaces and discuss anticipated multi-targeted approaches to overcome the identified barriers (Fiath et al., 2019, 2021).

We hope that by diagnosing the expected biotechnological challenges we are contributing to the field and will facilitate our peers to join in contributing to this challenging goal.

### Anticipated Barriers to Applying 3D Vertical Nanopillar Arrays for *in vivo* Intracellular Recordings

Based on current understanding, the most obvious barriers to applying the 3D vertical nanopillar technologies developed for *in vitro* studies to *in vivo* brain research are: the inflammatory encapsulating processes triggered by MEA platform implantation; the mechanical stability of the 3D vertical pillar platform during insertion of the implant; and the mechanics of the brain’s micro-pulsations.

The predominant literature in the field claims that the multicellular inflammatory foreign body response (FBR) of the brain to neuroprobe implantation leads to neuron degeneration, displacement of neurons away from the implant surface, and electrical insulation of the implant by the electrical resistance generated by the multicellular glia scar. These processes lead to deterioration of the quality and yield of the recorded extracellular FPs within days to weeks after implantation (Gulino et al., 2019; Wellman et al., 2019). Generations of researchers have attempted, with only marginal success, to overcome or ameliorate the FBR.

In recent years new neuro-engineering concepts of utilizing ultra-small and ultra-flexible MEA platforms have been developed and tested (Xiang et al., 2014; Fu et al., 2016; Luan et al., 2017; Zhao et al., 2017; Wei et al., 2018; Guan et al., 2019; Yang et al., 2019; Zhang et al., 2021). At the resolution of the confocal microscope, these implants indeed appeared to seamlessly integrate with mouse cortical parenchyma and appeared to “promote” neuronal cell bodies to reside close to the implant surface. Note, however, that despite the fact that the impedances of the microelectrode at the tips of the ultra-small and ultra-flexible platforms were similar to those of conventional large footprint implants (0.5–1 MΩ at 1 KHz) and despite the apparent seamless structural integration of these platforms with brain tissue, the recorded FP amplitudes were within the range of those recorded by “classical” large footprint implants that trigger FBR. As it is clear that the severity of the FBR with the ultra-small platforms was significantly reduced or even totally abolished (Zhang et al., 2021), these observations are inconsistent with the prevailing theory claiming that in the absence of a histological FBR the FP amplitudes should be larger (theoretically the field potential amplitude decreases in the brain’s extracellular space at a rate of 1/r*^x^* (where r is distance from the current source and x is in the range of 1 < x < 2, see Malaga et al., 2016; Michelson et al., 2018). This inconsistency suggested that there are other electrically insulating barrier(s) than the distributed FBR between the neurons and the electrodes. These barriers are present even when immunohistological observations demonstrate seamless integration of the implant (Huang et al., 2020; Sharon et al., 2021a,b). Understanding what mechanism(s) diminish the electrical coupling between neurons and MEA implants, even in the absence of immunohistological FBR, becomes essential for the development/application of intracellular recordings by 3D vertical nanopillars *in vivo.*

**FIGURE 2:**
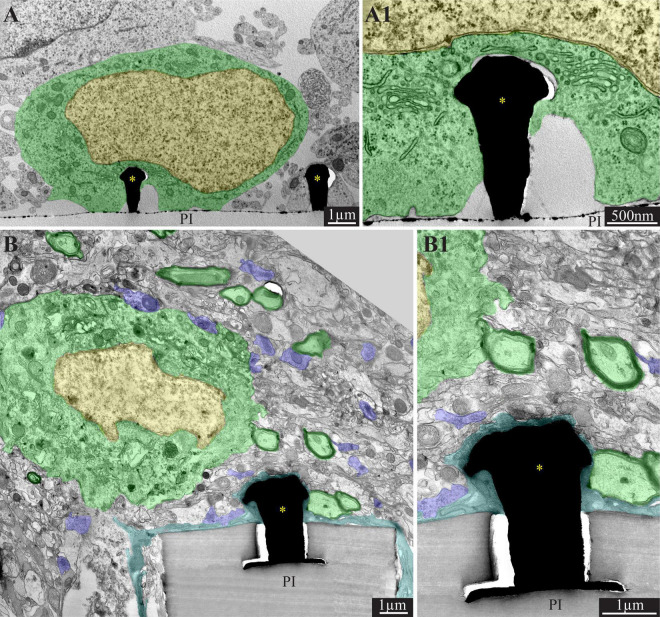
Comparison of the ultrastructural interfaces formed between gold mushroom-shaped microelectrodes (gMμE) and the cells around them in culture **(A,A1)** and under *in vivo* conditions **(B,B1)**. **(A)** In culture of primary rat neurons the neuron’s somata (green) engulf the gMμE “cap’s.” The narrow cleft formed between the neurons plasma membrane is free of other cells types **(A1)**. **(B)** In contrast, under *in vivo* conditions, the gMμE are insulated by thin layer(s) of dark microglia. In addition a network of regenerating neurites and astrocyte branches occupy the space between the neurons and the gMμE (see also schematics in Figure 1). Of interest is to note the remarkable regeneration of the parenchyma around the PPMP implant. The image shows a neuronal cell body (nucleus in yellow and cytoplasm in green) resides approximately a micrometer away from a gMμE and the PI platform’s surface. Myelinated axons (green surrounded by a black sheath) are distributed in the parenchyma in contact with the microglia that adheres to the platform. Unmyelinated neurites and synaptic structures (labeled purple) were identified (using large magnification of the image) by the presence of presynaptic vesicles. The remainder of the unmarked profiles are astrocyte branches, non-myelinated neurites and other cells. PI, polyimide ribs; gMμE, yellow asterisks. Note that an unmarked copy of this figure is presented as Supplementary Figure 1. Modified with permission from Sharon et al. (2021b).

In recent studies our laboratory proposed that microglia adhering to the surface of the MEA platform and the electrodes, rather than the FBR, form an insulating junction (Figures 1, 2; Huang et al., 2020; Sharon et al., 2021a,b). These insulating microglia were probably overlooked in earlier studies, as in the vast majority of studies designed to explore electrode/tissue structural interfaces, the implants were extracted from the brain tissue prior to thin sectioning for histological examination (Schultz and Willey, 1976; Moss et al., 2004; Grand et al., 2010; Marton et al., 2020). Implant extraction not only damaged the remaining tissue surrounding the void left by the removed probes but also destroyed the opportunity to examine the intimate relationships between the implant and the tissue.

We now briefly illustrate our observations and discuss their implications.

### Structural Examination of the Limitations on Integrating 3D Vertical Nanopillars With Brain Parenchyma

Structural assessment of the feasibility of using 3D vertical nanopillars for intracellular recording *in vivo* began by fabricating large footprint polyimide-based electrode platforms that can be thin-sectioned along with the surrounding tissue for immunohistological and electron microscope analysis (Figure 1D). Accordingly, we began by fabricating perforated polyimide (PI)-based MEA platforms (PPMP) decorated by dense gold mushroom-shaped non-functional microelectrode arrays. The design enabled us to merge sufficient platform stiffness and “sectioning ability” for both confocal and ultrastructural examinations (PI’s Young’s modulus is 2.5 GP and was experimentally shown to be compatible with brain tissue). Importantly, it was established that PI platforms can be thin-sectioned along with the surrounding brain tissue for immunohistological and ultrastructural studies (Mercanzini et al., 2007, 2008; Richter et al., 2013; Xie X. et al., 2014; Xie Y. et al., 2014; Boehler et al., 2017; Sharon et al., 2021b). To optimize the integration of the implant with the brain’s parenchyma we perforated the PI-based platform (Figure 1D, for fabrication details and dimensions see Sharon et al., 2021b). The selected microarchitecture was influenced by the “critical surface area hypothesis” of Seymour and Kipke (2007) and Skousen et al. (2011) who documented that the FBR induced by a solid-shank silicon MEA platform was more severe than that caused by a lattice architecture and the injectable mesh MEA platform developed in Dr. C. Lieber’s laboratory (Zhang et al., 2021).

Quantitative analysis of confocal microscope images of PPMPs implanted in rat motor cortices along with the intact tissue surrounding them established that the overall severity of the FBR (microglia, astrocytes and neurons) induced by the PI-based porous microarchitecture was smaller than that induced by solid implants with similar dimensions. In addition, the overall regenerative processes, judged by the density of neurites and neuronal cell bodies in the immediate vicinity of the implant, were good (Huang et al., 2020; Sharon et al., 2021a,b).

Of relevance to the subject of discussion, we noted that a fraction of overlooked individual microglia by the vast literature tightly and persistently adhere to the surface of the implanted platform (Figures 1E,F, 2B,B1). Consistent with earlier classical studies, PPMP implantation activates and increases the density of microglia and astrocytes in centripetal shells around the implant (for quantitative analysis see Huang et al., 2020; Sharon et al., 2021a). Whereas the astrocytes densities around the implant continued to increase for over 8 weeks after implantation, the overall increased microglia densities subsided and recover to almost control levels 4–8 weeks (Huang et al., 2020). It is of importance to note, however, that the density of microglia tightly adhering to the microelectrode surfaces persistently remains high. In contrast, astrocyte cell-bodies or branches rarely form intimate contact with the PPMP and electrodes throughout the 8 week period of studies.

A tentative explanation to account for the observation that a fraction of the microglia persistently remain adhering to the PPMPs while the overall microglia density recover to control is that the degree of substrate stiffness to which the microglia adhere leads to changes in the cell’s biological features (Moshayedi et al., 2014; Bollmann et al., 2015) or, alternatively, that these microglia represent infiltration of blood-borne immune cells through the breached BBB (Winslow and Tresco, 2010; Winslow et al., 2010; Saxena et al., 2013; Moshayedi et al., 2014; Ravikumar et al., 2014; Bedell et al., 2018). Whatever the source of the adhering microglia, we recently observed that, in contrast to the general microglia population distributed throughout the cortex, these adhering microglia are insensitive to the colony-stimulating factor 1 receptor (CSF1R) inhibitor PLX5622 (Sharon et al., 2021a). While PLX5622 administration leads to 95% elimination of the cortical microglia, the adhering microglia remain unaffected (Figure 1F).

Since the spatial resolution of confocal microscopy is insufficient to resolve the intimate structural interfaces formed between the implants and the brain parenchyma, we examined the interfaces formed between gMμE-PPMPs and rat cortical brain parenchyma under the electron microscope (Sharon et al., 2021b). TEM images revealed that the tissue around the gMμE-PPMP implant undergoes a remarkable regenerative process within 2–8 weeks of implantation. This culminates in regrowth of neurites toward the implant, myelination of the newly grown axons, the formation of structurally mature chemical synapses, the recovery of neuronal cell body densities in the close vicinity of the electrodes and cortical capillaries (Sharon et al., 2021b; Figures 2B,B1). The high resolution ultrastructural analysis complemented the confocal observations above, showing that along with the remarkable tissue regeneration neuronal cell bodies could be observed close to the PPMP and gMμEs and that microglia adhering to the gMμEs-PPMP surfaces form a physical barrier that mechanically isolates and electrically insulates the gMμE (Figures 2B,B1). On the other hand, astrocyte cell bodies or branches that extend between microglia rarely form direct contact with the implant surface or electrodes. Thus, for a period of approximately 8 weeks post-PPMP implantation (the longest observation period), the adhering microglia (but not astrocytes) appear to prevent the formation of a direct contact between axons or neuronal cell bodies and the gMμE (Figures 1E,F, 2B). Consequently, it appears that the insulating microglia are expected to impede engulfment of gMμEs by neurons and possibly direct piercing of neuronal membranes by other types of vertical nanopillars.

Another mechanism that is expected to impede the formation of direct contact between gMμEs or other 3D vertical nanopillars and the neuronal cell bodies is the regenerative extension of a dense network of thin neurites (≤1 μm) and astrocyte branches between the neuronal cell bodies and the electrodes (Figures 2B,B1). In addition, the low resistance of the extracellular spaces between the neurons and the vertical electrode is sufficient to shunt a large fraction of the current generated by propagating APs.

Examination of a large number of gMμE-PPMPs implanted in motor cortices of rats for periods of up to 8 weeks revealed that the gMμEs are stably anchored to the gold lines to which they were electroplated (Sharon et al., 2021b).

### The Identified Barriers and Possible Ways to Overcome Them

The emerging structural scenario described above demonstrates that implantation of large footprint gMμE-PPMPs into rat cortices initiates moderate FBR. This is associated with notable regenerative processes including neuritogenesis, myelination, synapse formation and recovery of neuronal cell body density near the implant. Nonetheless, in contrast to the configuration formed under *in vitro* conditions (Figures 2A,A1), three structural processes that are expected to impede the formation of direct contact between the gMμE and the neuronal cell bodies were identified: (a) the presence of PLX5622-insensitive microglia subtypes that adheres to the electrodes forming a high resistance barrier between them and the neurons (Figures 1C1,E,F, 2B). Theoretically, these microglia subtypes can be molecularly/pharmacologically characterized and eliminated either by reagents decorating the implant surface or applied systemically. (b) Regenerative neurites along with astrocyte branches that regrow and occupy the space between the neuronal cell bodies and the microelectrodes prevent the cell bodies from forming direct mechanical contact with the electrodes (Figures 1C2, 2B,B1). These branches (Figure 2B) are too small to effectively engulf the gMμE (diameter < 1 μm) and the neurite surface areas are too small to generate sufficiently large current to be sensed by the microelectrodes. Counterintuitively, it is conceivable that functionalization of the platform surface with molecules effectively inhibiting neurite outgrowth, for example NogoA (Fawcett, 2020), could locally prevent neuronal growth comes from navigating toward the electrodes. (c) Although the density of neuronal cell bodies recovers after gMμE-PPMPs implantation within a shell of 0–25 μm from the platform surface (Sharon et al., 2021b), the density of neuronal cell bodies in close contact (< 1 μm) with the platforms surface is too small to sufficiently increase the probability of engulfment of the gMμE by the neuron or of piercing of the neuron’s plasma membrane. The mechanisms underlying the recovery of neuronal cell body concentration in the vicinity of the electrodes were not investigated. Recent studies reveal active neurogenesis processes in the dentate gyrus of the hippocampus and the sub-ventricular zone. These niches serve as endogenous sources of neural precursor cells that can migrate and thus may potentially replace damaged or lost neurons at the site of injury. Attempts to develop neuro-engineering approaches to facilitate the migration of neuro-precursor cells toward “remote” sites of injury are underway (Bressan and Saghatelyan, 2020; Purvis et al., 2020). It is conceivable for the time being that implantation of 3D vertical nanopillar-based probes close to or at the sources of endogenous precursor neurons will reveal accelerated probability for a direct neuron/electrode interfaces. Note that the probability of successfully applying the above solution requires that all the targeted barriers are concomitantly addressed.

An alternative approach to bypassing the structural barrier is to implant 3D vertical MEA platforms that were electrically coupled to a layer of autologous neurons *in vitro* ahead of implantation (Adewole et al., 2019).

## Conclusion

This perspective identifies some of the challenging barriers to interfacing 3D vertical nano-probes with brain tissues *in vivo*. It is premature at this point to address additional challenges of the foreseen technology, such as the stability of the intracellular recordings by sharp nanopillars or the gMμEs configuration on the background of brain micro-pulsations. Nonetheless, an experimentally based roadmap to generate “dream neuroprobes” by merging the advantages of intracellular vertical nanopillars with contemporary *in vivo* MEA platforms for extracellular recordings is certainly feasible.

## Data Availability Statement

The original contributions presented in the study are included in the article/Supplementary Material, further inquiries can be directed to the corresponding author/s.

## Ethics Statement

The animal study was reviewed and approved by the Committee for Animal Experimentation at the Institute of Life Sciences of the Hebrew University of Jerusalem.

## Author Contributions

All authors listed have made a substantial, direct, and intellectual contribution to the work, and approved it for publication.

## Conflict of Interest

The authors declare that the research was conducted in the absence of any commercial or financial relationships that could be construed as a potential conflict of interest.

## Publisher’s Note

All claims expressed in this article are solely those of the authors and do not necessarily represent those of their affiliated organizations, or those of the publisher, the editors and the reviewers. Any product that may be evaluated in this article, or claim that may be made by its manufacturer, is not guaranteed or endorsed by the publisher.
