# Supplementary material for: Assessing the Feasibility of Developing in vivo Neuroprobes for Parallel Intracellular Recording and Stimulation: A Perspective
Source: Front Neurosci. 2022 Jan 25;15:807797. doi: 10.3389/fnins.2021.807797 (PMC8821521; doi:10.3389/fnins.2021.807797)
Supplement: Supplementary file 1 [file Table_1.DOCX]

**Supplementary**


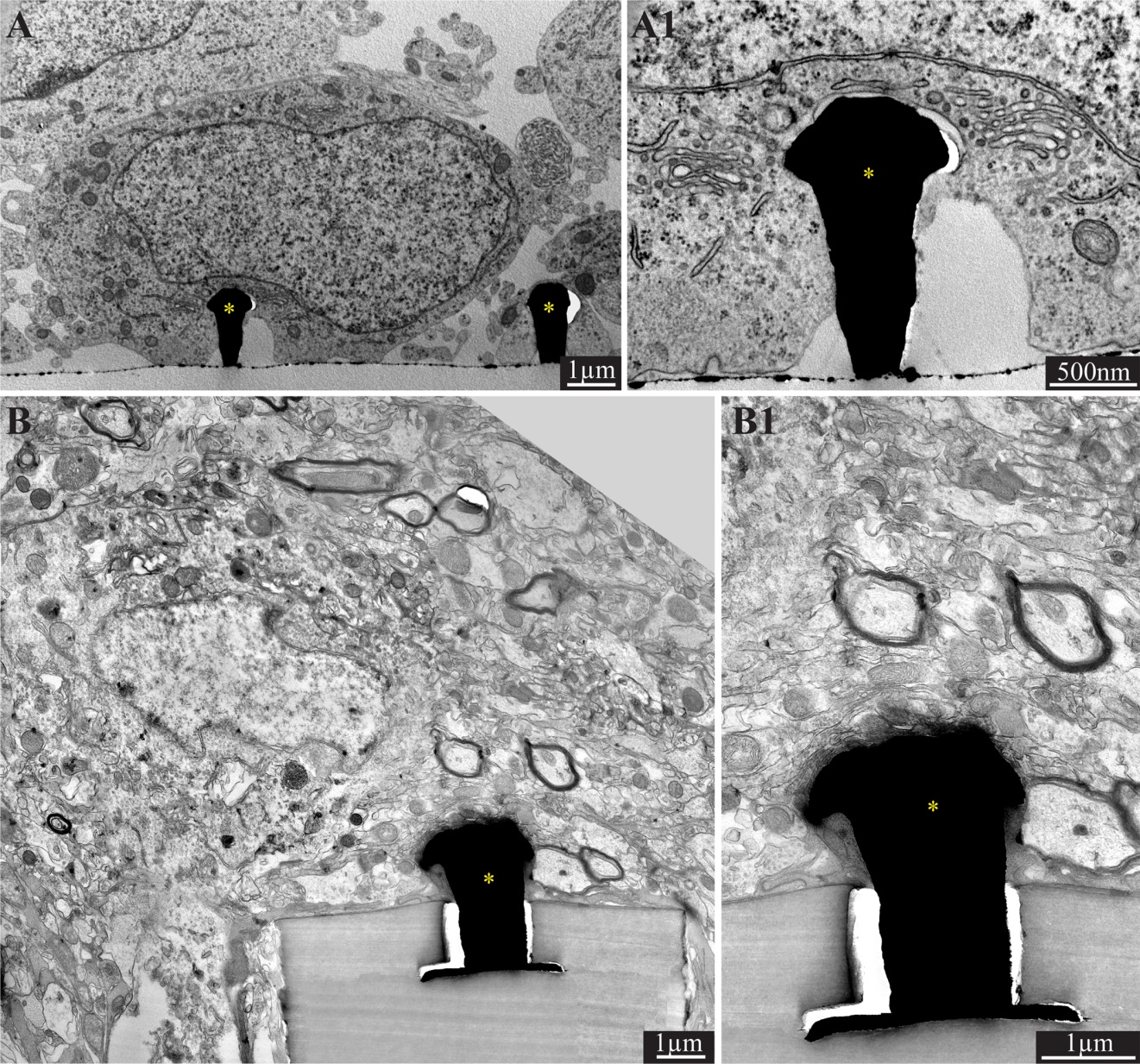
**Supplementary figure 1.** Comparison of the ultrastructural interfaces formed between gold mushroom-shaped microelectrodes (gMµE- asterisk) and the cells around them in culture (A and A1) and under in vivo conditions (B, B1). (A) In culture of primary rat neurons the neuron’s somata engulf the gMµE “cap’s”. The narrow cleft formed between the neurons plasma membrane is free of other cells types (A1). (B) In contrast, under in vivo conditions, the gMµE are insulated by thin layer(s) of dark microglia cytoplasm. In addition a network of regenerating neurites and astrocyte branches occupy the space between the neurons and the gMµE (see also schematics in Figure 1). Of interest is to note the remarkable regeneration of the parenchyma around the PPMP implant. The image shows a neuronal cell body (nucleus and cytoplasm) resides approximately a micrometer away from a gMµE and the PI platform's surface. Myelinated axons (surrounded by a black sheath) are distributed in the parenchyma in contact with the microglia that adheres to the platform. Unmyelinated neurites and synaptic structures were identified (using large magnification of the image) by the presence of presynaptic vesicles. Note a marked copy of this figure is presented as text figure 2.
